# Repeated Occurrence and Recurrence of Secondary Oral Solid Cancers after Hematopoietic Stem Cell Transplantation for Leukaemia: Long-Term Follow-Up

**DOI:** 10.1155/2022/9592077

**Published:** 2022-08-22

**Authors:** Takumi Takahashi, Miki Yamada, Keisuke Sawada, Nami Nakayama, Yosuke Iijima, Shunsuke Hino, Takahiro Kaneko, Norio Horie

**Affiliations:** ^1^Department of Oral and Maxillofacial Surgery, Saitama Medical Center, Saitama Medical University, Saitama, Japan; ^2^Department of Pathology, Saitama Medical Center, Saitama Medical University, Saitama, Japan

## Abstract

Hematopoietic stem cell transplantation (HSCT) is a common method for patients such as hematologic malignancies. However, HSCT generally has a higher risk of secondary solid cancer development. The aim of this study was to emphasize the need for lifelong follow-up of oral secondary solid cancer. The patient was a male who underwent HSCT for chronic myelogenous leukaemia at the age of 31 years. He underwent ten onsets on oral secondary solid cancers during his subsequent follow-up of more than 20 years. In conclusion, patients after HSCT require lifelong observation of oral secondary solid cancer, which may be accompanied by repeated new and recurrent occurrences.

## 1. Introduction

Hematopoietic stem cell transplantation (HSCT) is a vital therapeutic method for most patients who have hematologic malignancies such as leukaemia and non-malignant disease such as aplastic anaemia. HSCT has increased steadily over the past three decades worldwide [1–3], and has greatly improved the patient's long-term survival rate. However, HSCT generally has a higher risk of secondary solid cancer development, which became an important cause of late mortality, regardless of the stem cell source [2]. Among the secondary solid cancers, the high-risk cancer includes oropharyngeal cancer, oesophageal cancer, colon cancer, skin cancer and brain/nervous system cancer [1]. In these, oropharyngeal cancer is most frequent, and the incidence rate of oropharyngeal cancer after HSCT ranges from 32 to 92 per 100,000 person-years with a 7- to 16-fold higher risk than the general population [4]. The risk of secondary solid cancers begins to increase around 10 years after transplantation. The rate of secondary solid cancers was 1–6% at 10 years and 6–13% at 15 years [1, 2, 4]. Limited to oral secondary solid cancers, the time from HSCT to the onset of secondary solid oral cancers have been 1–17 years or more, and Mawardi et al. described that mean time was 8 years [5, 6]. The risk of secondary solid cancers continues even 20 years later and the risk of developing of oropharyngeal cancer continues to life-long [1, 4].

Although a relatively large number of oral secondary solid cancers have been reported, including statistical observations, most were merely reporting of first onset and have a short follow-up period [1, 7]. Oral secondary solid cancers can occur and recur in various sites of the oral cavity repeatedly over a long period of time. However, there have been a few reports of long-term follow-up, looking at how often the secondary cancers occur and recur during that time [6, 8–10].

In this study, we report a case of oral secondary solid cancers with ten times repeated occurrence and/or recurrence, which has been followed up for more than 20 years after undergoing HSCT at the age of 31 years in order to emphasize the need for long-term observation of the onset of oral secondary solid cancer.

## 2. Case Report

In April 2019, a 51-year-old man visited the oral and maxillofacial surgery clinic to continue follow-up of oral examination after HSCT. The patient was previously diagnosed with chronic myelogenous leukaemia in 2000. In September 2000, the patient underwent an allogeneic HSCT from his human leukocyte antigen–matched sister. Immediately after the transplantation, graft-versus-host disease (GVHD) of oral mucosa appeared. However, no prominent symptoms of GVHD other than the oral cavity were observed, and immunosuppressive therapy associated with GVHD was not performed. In addition, no local therapy was given.

After that, the patient could continue to follow up at the previous oral surgery clinic for oral GVHD and underwent surgery for secondary solid cancers of right buccal mucosa and left mandibular gingiva, both of which were diagnosed as squamous cell carcinoma (SCC) in February 2012; left mandibular gingiva in July 2012 (SCC); left mandibular gingiva in September 2012 (SCC); and right upper lip (SCC) in July 2013. Subsequently, biopsies were performed on the left upper lip in November 2017 and on the right side of the dorsum of the tongue in December 2017, both of which were negative for malignancy. After that, regular observations were continued, but due to work, our department took over the flow-up.

His medical history other than that associated with chronic myelogenous leukaemia included hypertension, hyperuricemia, chronic kidney disease, diabetes, and dyslipidemia. Current medications included omega-3-acid ethyl esters (hyperlipidemia), atorvastatin calcium hydrate (hypercholesterolaemia), amlodipine besilate (hypertension), perindopril erbumine (hypertension), febuxostat (hyperuricemia), and insulin aspart (diabetes).

**Figure 1:**
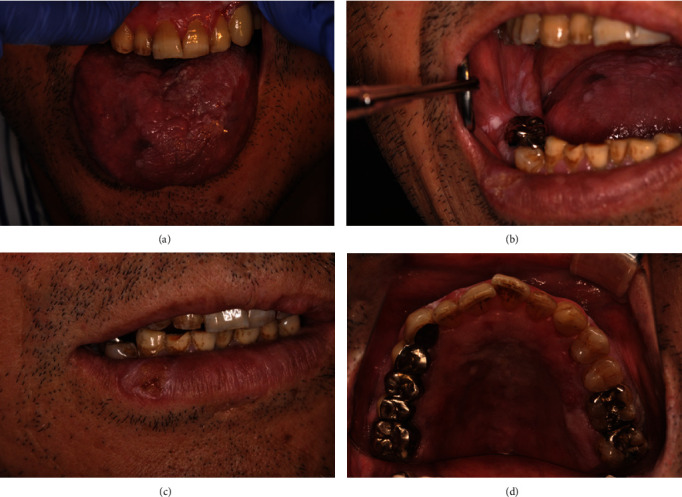
Clinical photographs (April 2019), (a) Erythema is observed on the right side and left margin of the tongue, and exophytic masses are found from the center to the left side of the dorsal surface. The left maxillary anterior gingiva shows whitish change. (b) The right buccal mucosa and pharyngeal mucosa shows lichenoid striae with erythema, in which white slightly raised lesions are observed, and white plaque is found posterior to the molar. (c) The right side of lower lip, a white lesion with a diameter of 7 mm, with an indistinct border, no induration, is observed. (d) Most of the palate shows erythema. A relatively clear white ridge is observed on the right molar (Mirror image).

On examination, erythema was observed on the right side and left margin of the tongue, while on the dorsal surface, exophytic masses were found. The left maxillary anterior gingiva showed whitish change (Figure 1(a)). The right buccal mucosa and pharyngeal mucosa showed lichenoid striae with erythema, in which white slightly raised lesions were observed, and white plaque was found posterior to the first molar (Figure 1(b)). On the right side of lower lip, a white lesion with a diameter of 7 mm, with an indistinct border and no induration, was observed (Figure 1(c)). Most of the palate showed erythema. A relatively clear white ridge was observed on the right molar (Figure 1(d)).

**Figure 2:**
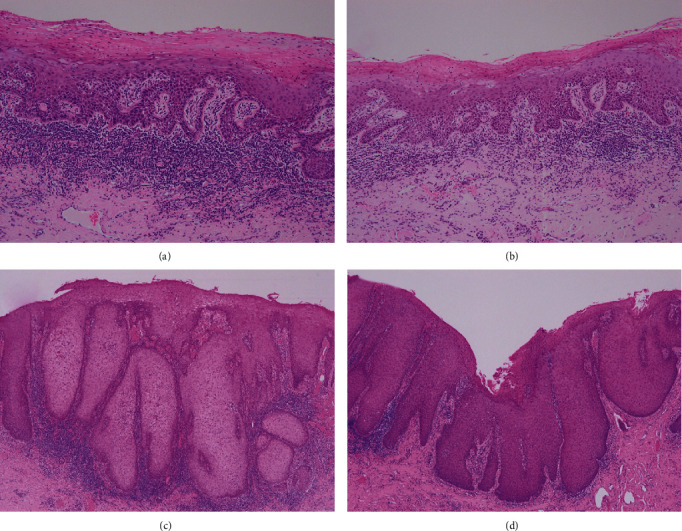
Photomicrographs of surgical specimens. (a) Specimen of tongue showing severe dysplasia. (b) Specimen of the right buccal mucosa showing severe dysplasia. (c) Specimen of the right side of lower lip showing squamous cell carcinoma. (d) Specimen of the right palatal gingiva showing carcinoma in situ (hematoxylin-eosin stain—original magnification ×100; (a and b), ×40; (c and d) ×40).

**Figure 3:**
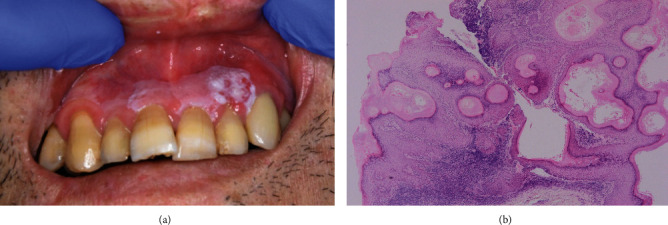
Clinical photographs (April 2021). (a) Compared to the April 2019 photograph, white lesions in the maxillary anterior gingiva have clearly developed and plaque formation is observed. (b) Surgical specimen shows severe dysplasia (hematoxylin-eosin stain, original magnification ×40).

After the biopsies, in June, surgical resections were performed. The diagnosis of the left dorsal surface of the tongue and the right buccal mucosa was severe dysplasia, and lower lip was SCC, and the left maxillary palatal gingiva was carcinoma in situ (Figures 2(a)–(d)). In addition, the specimens were immunostained with anti-p16 antibody to search for human papillomavirus (HPV) infection. Histopathologically, HPV infection was negatively diagnosed. In April 2021, a white lesion in the maxillary anterior gingiva became prominent, and a biopsy revealed severe dysplasia, resulting in surgical procedure (Figure 3). To date, no relapse of leukaemia has been observed. The patient has seen recurrence and the development of new lesions, but so far, the early response has been successful. Strict follow-up needs to be continued in the future.

## 3. Discussion

With improved clinical outcomes of hematologic diseases performed by HSCT, there is rising interest in secondary solid cancers after HSCT. Among them, oropharyngeal region is the most prominent high-risk organ of development of secondary solid cancers [1, 7]. In this study, we presented a case of patient with 10 times repeated new and recurrent oral secondary solid cancers who had been followed up for more than 20 years after HSCT.

Skin and oropharyngeal secondary solid cancer is predominantly squamous cell carcinoma [2]. Various risk factors have been reported for the development of secondary oropharyngeal solid cancers. In these risk factors, chronic GVHD is most important [1, 2, 4, 7]. GVHD is a major cause of morbidity and mortality in patients who undergo HSCT [11]. GVHD is divided into acute and chronic GVHD. Acute GVHD is a reaction of donor immune cells against host tissues, and the three main tissues that acute GVHD affects are the skin, liver, and gastrointestinal tract. Chronic GVHD is a syndrome of variable clinical features resembling autoimmune and other immunologic disorders, and manifestations of chronic GVHD may be restricted to a single organ or site or may be widespread, with profound impact on quality of life. After HSCT, GVHD is estimated to occur in 30–40%, and 25–80% of chronic GVHD patients present with oral symptoms [11]. Mawardi et al. described that 96% of patients with oral secondary solid cancers affected oral chronic GVHD [5].

The symptoms of oral acute GVHD and chronic GVHD may overlap. Diagnostic features of oral chronic GVHD include lichen planus-like changes, characterized by hyperkeratotic white lines and lacy-appearing lesions on the oral mucosa, and distinctive features of chronic GVHD include xerostomia (dryness), mucoceles, mucosal atrophy, ulcers, and pseudomembranes [12]. Symptoms that are common to both acute and chronic GVHD include gingivitis, mucositis, erythema, and pain [12].

The clinical and histological features of chronic GVHD are very similar to those of oral lichen planus. In both diseases, the immune system plays a primary role in the pathogenesis, and the same mechanisms are considered to be associated with malignant transformation. Clinically, it may be difficult to distinguish the secondary solid cancers from chronic GVHD with lichen planus-like changes. The most frequent clinical features presented at diagnosis of the solid cancers were described to be plaques (50%), exophytic masses (39%), and ulcers (28%) [5]. The median interval from the diagnosis of oral chronic GVHD to the diagnosis of oral secondary cancer was six years [5].

Other risk factors of the oropharyngeal cancers included reduced-intensity conditioning regimens associated with HSCT, autologous HSCT and hereditary disorders such as Fanconi anaemia in paediatric patients. Limited to oral cancer, long-term immunosuppressive therapy associated with chronic GVHD ≥24 months, conditioning regimens with limited field irradiation, and male gender were risk factors [2, 7]. In immunocompromised patients, oncogenic viruses such as HPV may contribute to oropharyngeal SCC [7]. In this case, as a result of immunostaining, HPV infection was ruled out. It was described that there has been no association between the incidence of secondary solid cancers and donor type or human leukocyte antigen [1, 4].

Secondary oral solid cancers can occur at any site in the oral cavity, but Mawardi et al. stated that the occurrence at the tongue was 56%, being the most predominant site, followed by buccal mucosa, 39% [5]. It needs to be emphasized that secondary oral solid cancers may appear multifocal or recur. Mawardi et al. described that multifocal oral cancers were found in 28% of cases, and localized recurrence was observed in 44% of their cases [5]. However, detailed reports of how many new occurrences and recurrences occurred during the observation period have been rare. To the best of our knowledge, there have been four cases showing the multiple onsets in chronological order, including new occurrences and recurrences, 3 cases with 2 onsets, and 1 case with 6 onsets (Table 1) [6, 8–10]. In the present case, there have been ten onsets in almost 10 years, and there have been no reports of follow-up of oral condition for more than 20 years in detail. According to the concept of field cancerization, secondary oral solid cancers are stated to show repeated onset of new lesions and recurrence [5]. It is important to know that secondary oral solid cancers may develop more than 10 years after HSCT and that they occur repeatedly over the years, in order to examine patients who have undergone HSCT.

As for follow-up, it is predicted that the secondary oral solid cancers will occur for a lifetime, so regular and uninterrupted scrutiny is necessary. Screening every six months may be considered for patients at high risk for developing oropharyngeal cancer [7]. Evaluation for premalignant lesions or oral cancer by a dentist or an oral surgeon is extremely important. The development of secondary solid cancers was likely to be influenced by GVHD-affected sites [1, 5]. Therefore, scrutiny of patients with chronic GVHD is important.

## 4. Conclusion

This study reported a patient with repeated new and recurrent secondary oral solid cancers who were followed up for more than 20 years after HSCT. The number of leukaemia survivors with HSCT is expected to increase in the future. In particular, there has been a dramatic increase in the number of transplant recipients who are more than 50 years of age at transplant over the past decade [1]. Dentists and oral surgeons should keep in mind that recipients of HSCT such as survivors of leukaemia need lifelong follow-up for new and recurrent oral secondary solid cancers.

## Figures and Tables

**Table 1:** Cases of multifocal and recurrence of secondary oral solid cancers after hematopoietic stem cell transplantation.

	Primary disease	Age at the time of HSCT (gender)	Age at the time of initial oral secondary cancer	Site of oral secondary cancer including new occurrences and recurrence (diagnosis^a^)	Time after HSCT
Abdelsayed et al. [8]	ALL	22 (M)	24	Left buccal mucosa (CIS)	2 y 5 m
				Left buccal mucosa (mild-to-moderate dysplasia)	2 y 10 m
Kano et al. [9]	CML	33 (F)	45	Tongue (SCC)	12 y 6 m
				Oral cavity (SCC)	13 y 7 m
Zhang et al. [10]	AML	49 (M)	54	Lower lip (CIS)	5 y 1 m
				Lower lip (CIS)	5 y 6 m
Weng et al. [6]	AML	37 (M)	42	Left buccal mucosa (SCC)	5 y
				Right maxillary gingiva (SCC)	5 y
				Left buccal mucosa (SCC)	5 y 3 m
				Right maxillary gingiva (SCC)	7 y
				Right maxillary gingiva (mild dysplasia)	7 y 6 m
				Right buccal mucosa (SCC)	
Takahashi et al. (present study)	CML	31 (M)	44	Right buccal mucosa (SCC)	11 y 5 m
				Left mandibular gingiva (SCC)	
				Left mandibular gingiva (SCC)	11 y 10 m
				Left mandibular gingiva (SCC)	12 y 0 m
				Right upper lip (SCC)	13 y 10 m
				Left dorsal surface of tongue (severe dysplasia)	19 y 1 m
				Right buccal mucosa (severe dysplasia)	
				Right lower lip (SCC)	
				Left maxillary posterior palatal gingiva (CIS)	
				Left maxillary anterior gingiva (severe dysplasia)	21 y 1 m

^a^Including not only squamous cell carcinoma but also carcinoma in situ and dysplasia.

ALL, acute lymphoblastic leukaemia; AML, acute myelogenous leukaemia; CIS, carcinoma in situ; CML, chronic myelogenous leukaemia; HSCT, hematopoietic stem cell transplantation; SCC, squamous cell carcinoma.
